# Adherence of *Streptococcus Mutans* to Microhybrid and Nanohybrid Resin Composites and Dental Amalgam: An In Vitro Study

**Published:** 2017-11

**Authors:** Fariba Motevasselian, Ensieh Zibafar, Esmail Yassini, Mansoreh Mirzaei, Naser Pourmirhoseni

**Affiliations:** 1 Assistant Professor, Dental Research Center, Dentistry Research Institute, Tehran University of Medical Sciences, Tehran, Iran; Department of Restorative and Aesthetic Dentistry, School of Dentistry, Tehran University of Medical Sciences, Tehran, Iran; 2 Assistant Professor, Department of Medical Parasitology and Mycology, School of Public Health, Tehran University of Medical Sciences, Tehran, Iran; 3 Professor, Department of Restorative and Aesthetic Dentistry, School of Dentistry, Tehran University of Medical Sciences, Tehran, Iran; 4 Associate Professor, Department of Restorative and Aesthetic Dentistry, School of Dentistry, Tehran University of Medical Sciences, Tehran, Iran; 5 Dentist, Private Practice, Tehran, Iran

**Keywords:** Bacterial Adhesion, Dental Amalgam, Dental Restoration, Resin Composite, *Streptococcus Mutans*

## Abstract

**Objectives:**

*Streptococcus mutans* (*S. mutans*) is a cariogenic microorganism. The restorative materials which harbor a biofilm with high levels of *S. mutans* can accelerate the occurrence of dental caries. The purpose of this study was to evaluate the influence of different restorative materials on *S. mutans* colonization in a simple in-vitro biofilm formation model.

**Materials and Methods:**

Thirteen discs of each material (nanohybrid resin composite, microhybrid resin composite, and amalgam) were prepared, polished, and sterilized in a gamma radiation chamber. The saliva-free specimens were exposed to the *S. mutans* bacterial suspension (0.5 McFarland) and were incubated for 4 hours. Afterwards, the specimens were rinsed and sonicated in normal saline. 10μl of the obtained suspension was cultured in a sterile blood agar medium. After 24 hours, the number of colony forming units (CFU) of *S. mutans* was counted. A sterility test control was considered for each group of materials. The data were analyzed by one-way ANOVA at 5% significance level.

**Results:**

The means and standard deviations of the logarithmic values of the colonies on the surfaces of amalgam, microhybrid, and nanohybrid resin composites were equal to 3.76±0.64, 3.91±0.52 and 3.34±0.74, respectively.

**Conclusions:**

There were no significant differences between the restorative materials in terms of *S. mutans* adhesion rate. The evaluated resin composites showed comparable numbers of CFUs, which could imply the importance of the polishing procedures.

## INTRODUCTION

The biofilm is developed on oral surfaces by microbial species covered in a self-produced medium of extracellular polymeric substances mediating microorganism adhesion to different substrates [1]. The adhesion of bacteria to teeth and dental restorative materials can cause dental caries [2] and other oral diseases [3]. Among the species present in a cariogenic biofilm, *Streptococcus mutans* (*S. mutans*) is recognized as one of the main cariogenic bacteria [4]. Therefore, the evaluation of the adhesion and colonization of *S. mutans* on restorative materials is important for improving the clinical performance and success rate of these restorations [5, 6]. Currently, many different restorative materials are available. For many years, amalgam has been the main restorative substance [7]. Although the use of dental amalgam has declined, it is still the most widely used direct restorative material for load-bearing posterior restorations [8]. The popularity of dental resin composites is increasing [7] due to their outstanding esthetics and the advantages of the adhesive technology [9]. Several manufacturers have provided a wide range of resin composites [10], and the current differences among these materials are mainly related to their inorganic filler components, which might influence their properties [11, 12]. Nanohybrid resin composites have recently been introduced to the market in an endeavor to provide a polishable material with a good polish retention [13]. Nanohybrid resin composites contain a combination of nanomeric and conventional fillers [14], similar to microhybrid resin composites [13]. Therefore, the distinction between microhybrids and nanohybrids is not always obvious [15]. The surface properties of restorative materials are critical for their success since they mediate the interaction of these materials with the oral environment, including bacterial accumulation [16, 17]. These surface features include the chemical composition of the material, the nature of the substrate [18] and the surface roughness [17, 19]. It has been shown that the particle size of resin composites has a significant impact on the surface roughness of these materials [20]. The correlation between the surface roughness of resin composites and biofilm formation has been previously reported [21, 22]. However, little is known about bacterial adherence to nanohybrid resin composites. There are multiple in-vitro biofilm formation models, from simple ones with a single bacterium to complex multispecies designs [23]. Oral streptococci have been frequently used in caries models [24]. Streptococcal adhesion to a substrate is often mediated by a conditioning film such as artificial saliva or human saliva [23]. The formation of *S. mutans* biofilms has been simulated in a monospecies model without prior salivary pellicle formation [25], and it has been stated that *S. mutans* bacteria more effectively adhere to the surfaces which are not covered by saliva [26], which might justify its selection for the monospecies biofilm model. Currently, there is no distinctive information on comparing the bacterial colonization on microhybrid and nanohybrid resin composites. Therefore, the present in-vitro study was designed to determine the colonization of *S. mutans* on saliva-free surfaces of three restorative materials, including nanohybrid and microhybrid resin composites and dental amalgam, in a simple biofilm formation model. The null hypothesis was that the colonization rates of *S. mutans* are significantly different on the surfaces of different restorative materials.

## MATERIALS AND METHODS

Three commercial restorative materials, including nanohybrid resin composite (Filtek^TM^ Z250XT, 3M ESPE, St. Paul, MN, USA), microhybrid resin composite (Filtek^TM^ Z250, 3M ESPE, St. Paul, MN, USA), and dental amalgam (Tytin^TM^, Kerr Corp., CA, USA) were tested in this study (Table 1).

**Table 1.** Specifications of the materials used in this study

**Material**	**Manufacturer**	**Classification**	**Composition**
Tytin^TM^	Kerr Corp., CA, USA	High-copper spherical amalgam	Powder: 59% silver (Ag), 28% tin (Sn), 13% copper (Cu) Liquid: 42.5%wt mercury (Hg)
Filtek^TM^ Z250	3M ESPE, St. Paul, MN, USA	Microhybrid composite	Organic matrix: BIS-GMA, UDMA, BIS-EMA, PEGDMA and TEGDMA Inorganic filler: Zirconia and Silica particles≤3μm Non-agglomerated/non-aggregated silica particle: 20nm
Filtek^TM^ Z250XT	3M ESPE, St. Paul, MN, USA	Nanohybrid composite	Organic matrix: Bis-GMA, UDMA, Bis-EMA Inorganic filler: Zircon and Silica particles: 0.6μm (0.01–3.5μm)

### Preparation of specimens:

Thirty-nine disk-shaped specimens (13 for each material) with a diameter of approximately 5mm and a height of approximately 1mm were fabricated. The materials were formed in a calibrated circular plexiglass mold. A clean glass slab was placed beneath this mold for support and to ensure proper condensing of the materials. After the insertion of the resin composites into the mold, the surface was covered with a celluloid tape to minimize the formation of an oxygen-inhibited layer, and each side was light-cured for 40 seconds using a light-curing device (Bluephase G2, Ivoclar Vivadent, Mississauga, Canada) with the light intensity of 1200 mW/cm^2^ at a distance of about 1mm from the surface. All the specimens were then removed from the mold, were evaluated for visible surface defects, and were polished with moderate and fine Sof-Lex polishing discs (3M ESPE, St. Paul, MN, USA) using a low-speed handpiece. The amalgam was also condensed into the mold. After 24 hours, the specimens were burnished and polished with the use of the amalgam polishing kit (Kerr Corp., CA, USA). The disk-shaped samples were then washed in distilled water and were sterilized in a 20-kGy gamma radiation chamber (cobalt 60) for 6 hours [27].

### *S. mutans* adhesion assay:

A bacterial suspension of a reference strain of *S. mutans* (PTCC 1683) with a concentration equal to 0.5 McFarland turbidity (10^8^ bacteria/ml) was prepared in sterilized normal saline. Each of the disks was aseptically placed at the bottom of a 24-well plate. Afterwards, 350μl of sterilized normal saline and 350μl of the bacterial suspension were poured into each well. For each group of materials, a negative control (sterility test control) was designated, consisting of the disk-shaped specimens immersed in 700μl of sterilized normal saline, which were also placed in the wells. Then, the specimens were incubated in 5% CO_2_ at 37 °C for 4 hours. This incubation period was chosen since complete oral biofilm formation during 2 to 4 hours has been previously described [5]. The specimens were then removed and were washed three times with sterile normal saline (each time for one minute) in order to remove the non-adhered cells [5]. Afterwards, the samples were placed in wells filled with sterile normal saline and were sonicated (Tecna 3, Tecno-Gaz dental and medical equipment, Parma, Italy) for 6 minutes to disperse the adhered cells in the solution [5]. 10μl of the obtained suspension was linearly seeded on sterile blood agar culture medium (Darvash Co. Ltd, Tehran, Iran). The culture plates were then incubated at 37°C for 24 hours. This process was also performed on the negative control disks to rule out any contamination. After the incubation period, the number of bacteria in the broth was counted. Since the colony count in most plates was numerous, the counting was performed by the Olysia BioReport imaging software program (Olympus, Tokyo, Japan).

### Statistical analysis:

Data were subjected to statistical analysis by means of one-way ANOVA using SPSS version 22 software program (IBM Co., Chicago, IL, USA). The level of significance was set at 5%.

## RESULTS

**Fig. 1:**
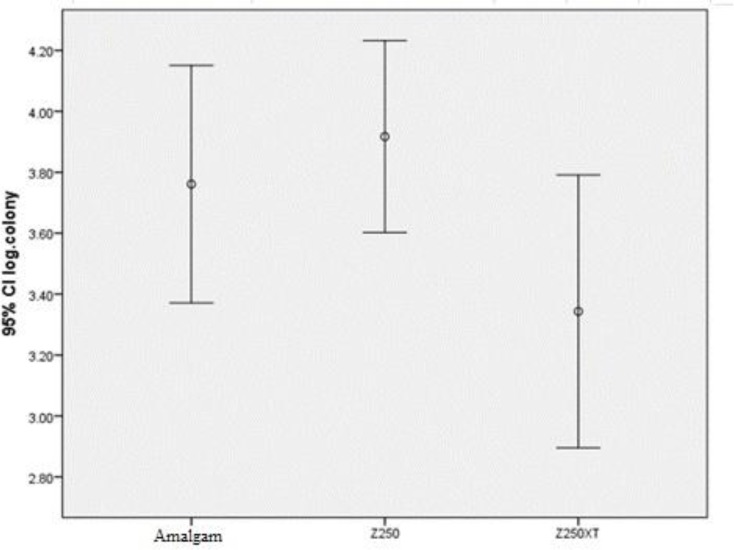
The mean and 95% confidence interval (CI) of the logarithmic values of the colony forming units (CFU) of *Streptococcus mutans* (*S. mutans*) on the evaluated restorative materials.

The means, standard deviations and logarithmic values of the number of colony forming units (CFU) on the restorative materials are presented in Figure 1. The tested materials showed a similar adhesion of *S. mutans*, and pairwise comparisons of the materials also showed no statistically significant differences in terms of bacterial adhesion (P=0.076).

## DISCUSSION

In addition to the proper technique, different physical, chemical and biological properties of the restorative material also influence the long-term success of a dental filling [28]. According to several reviews, it has become obvious that bacterial adhesion is a highly complex process [29, 30]. The biofilm formation models are commonly used to help us understand this complex process and the related influential factors [29]. In the present study, bacterial adhesion was assessed only for few hours, similar to the duration usually adopted in a monospecies biofilm model [5]. The results of this study did not confirm our original hypothesis since *S. mutans* did not demonstrate different adhesion rates on the tested materials. Several studies have assessed the biofilm formation on different restorative materials and have reported similar biofilm formation rates on composite resins and amalgam [31, 32]. It has been stated that resin composites are suitable for bacterial adhesion and might cause more plaque accumulation in comparison with the materials which are harmful to the adhering bacterial population [33]. However, despite the antimicrobial properties of the heavy metals released from the amalgam [34, 35], biofilm formation on this material was not significantly different from that on the resin composites in the current study. A quantitative analysis of the biofilm structure accumulated in-situ on different restorative materials showed that the developed biofilms were structurally similar, irrespective of the type of restorative materials [31]. The authors proposed that different ions released from the materials have not been able to significantly change the amount of the accumulated biofilm [31]. This might be due to the production of exopolymeric substances (EPS), which immobilize the ions [36]. Surface roughness is another factor reported in the literature that may have an influence on the adhesion and retention of oral bacteria [17].

Bollen et al [37] suggested a threshold surface roughness for bacterial retention (Ra=0.2μm) below which no further reduction in bacterial accumulation could be expected. However, an increase in the surface roughness above this threshold resulted in a simultaneous increase in plaque accumulation [37]. Polishing can minimize the critical threshold of surface roughness [38]. In the current study, all the specimens were polished to closely simulate the clinical conditions; this might have decreased the surface roughness to below the mentioned threshold; therefore, the surface roughness did not influence the *S. mutans* accumulation on the tested materials. In addition, the current results indicated that the behavior of Filtek^TM^ Z250XT nanohybrid resin composite in terms of *S. mutans* colonization was not statistically different from that of Filtek^TM^ Z250 microhybrid resin composite. The different bacterial adhesion rates on resin composites can be related to the particle size, hardness and chemical composition of the resin matrix [25].

Nanohybrids are hybrid resins with nanofillers to fill the gaps between larger particles [14]. Microhybrids also contain a small portion of nano-sized particles [15]. On average, the particle size is typically below 1 micron; however, it is above 0.2 microns in these two types of resin composites [15]. It is worth mentioning that both Filtek^TM^ Z250 and Z250XT resin composites contain zirconia and silica particles with a similar average filler size [15], which might suggest identical surface parameters that resulted in a similar *S. mutans* colonization rate. Furthermore, these resin composites present the same organic matrix components, except that the polyethylene glycol dimethacrylate (PEGDMA) has substituted some of the triethylene glycol dimethacrylate (TEGDMA) in Filtek^TM^ Z250 to moderate the shrinkage of Filtek^TM^ Z250XT resin composite [15]. Therefore, the similar amount of *S. mutans* adherence on these two types of composite resins might be associated with the similar filler fraction and resin components. In an investigation by Hansel et al [39], no difference in the adhesion of different bacterial strains was observed between the two evaluated resin composites with a similar composition of resin monomers. In a study by de Moraes et al [13], the properties of a nanohybrid composite resin were evaluated in comparison with nano-filled and microhybrid composite resins. They indicated that the behavior of nanohybrid resin composites is similar to that of microhybrid resin composite [13]. The results of the present study should be interpreted by considering its limitations, including its in-vitro nature and simulation of single-species biofilm formation without previous salivary pellicle formation. Further investigations on artificial mouth model systems, which simulate the acquired pellicle formation in multispecies biofilm formation models, are highly suggested to achieve restorative surfaces with a low bacterial colonization rate.

## CONCLUSION

The dental amalgam, which is known to have anti-adherent properties, did not show any significant difference in the bacterial adhesion compared to the resin composites. Filtek^TM^ Z250XT nanohybrid and Filtek^TM^ Z250 microhybrid resin composites showed similar behaviors in terms of *S. mutans* colonization in a simple biofilm formation model, which may indicate the similar surface properties of these two types of resin composites. Within the limitations of this study, the moderately finished and polished surfaces of the evaluated materials showed a similar susceptibility to bacterial adhesion, which emphasizes the importance of following the minimum requirements of the polishing procedures.
